# Healthy working environments are essential, but what exactly is HWE policy and who would be responsible for its success?

**DOI:** 10.1016/j.puhip.2026.100861

**Published:** 2026-09-29

**Authors:** Paul Cairney, Eli Auslender, Lukas Fuchs

**Affiliations:** School of Law, Politics and Social Sciences, University of Stirling, UK

## Abstract

A fundamental shift towards healthy working environments is essential, and it requires meaningful collaboration between employers, workers, and policymakers. While this argument may seem clear in public health research and practice, its implications for policy are profoundly unclear. This lack of collective clarity and purpose will undermine meaningful progress. We identify the need to address three questions: what exactly is HWE policy, what policy instruments should be adopted, and who would be responsible for their design, delivery, and coordination? We use the example of EU policy innovation to highlight the disconnect between two current answers: a *top-down* approach to obliging and enforcing minimum standards, and a *bottom-up* approach to going beyond those standards. The overall approach is incomplete: an ambitious and well-supported strategy does not produce a substantive impact on public and private action. We have not witnessed the ‘fundamental shift’ in policy envisaged by this Special Issue of Public Health in Practice.

## 1 Introduction

This journal's Special Issue on *Work Welfare and Health* welcomes the call for a ‘fundamental shift towards a *healthy working lifecycle*, advocating shared responsibility between employers, employees and government’. It argues that ‘the implications for multi-disciplinary public health practice are clear’.

In this Policy and Debate article, we argue that the implications for policy and policymaking are *not* clear, and this lack of clarity helps to explain a lack of meaningful progress. While such policy ideas and agendas seem self-evident to public health scholars and practitioners, they lack the same meaning outside of this profession, and struggle to gain traction in policy and practice. The solution is more collective clarity and purpose regarding what the policy would be, the key means to support it, and who would be responsible for its success. Otherwise, healthy work advocates would be supporting the same vague ambitions but contributing to different approaches and expectations on what can happen.

To demonstrate, we draw on new research on *healthy working environments* (HWE) policy in the EU for the Horizon Europe project WAge, which is part of the WISEWORK cluster (see Funding statement). Our aim is to foster evidence-informed policy by engaging with policymakers, employees, and employers to develop useable research and technologies to support the long-term physical and mental health of workers. In that context, ‘healthy working environment’ sums up a win-win-win ambition to boost: good physical health, high satisfaction, and a sense of purpose among workers; the productivity of workplaces via the reduction of absence due to ill-health, disability, or disengagement; and, the economic productivity of a country or region.

This HWE ambition is aspirational, innovative, and a source of potential international lessons. However, it is also vague; it lacks a concrete strategy to support well-specified actions and a workable theory of change. Hence, we identify the three questions that need clearer answers when policymakers seek to learn how to translate HWE ambitions into reality: (1) what exactly is HWE policy, (2) what policy instruments should be adopted in its name, and (3) who or what would be responsible for influencing, making, delivering, or coordinating these instruments?

## 2 What exactly is HWE policy?

‘Public policy’ is notoriously difficult to define. It is an umbrella term that can include statements on desired policy aims and outcomes, a wide range of policy instruments used by governments to produce outcomes (from taxes and regulations to guidance and voluntary agreements), and governmental and non-governmental contributors to policy outcomes (such as the practices of workplaces responsible for delivering policy or meeting required standards). [1] Hence, the first problem is to identify what policy actors mean by HWE policy, and the extent to which they go beyond vague aspiration to identify which instruments should be used to produce which desired outcomes.

International organisations and researchers tend to focus on aspirational aims and desired practices and outcomes when they (1) describe *healthy workplaces*, *working environments*, or *lifecycles* and (2) recommend integrated and coherent policies that recognise the value of workplace health to public health, employment, and economic aims. [[2], [3], [4]] They seek to widen the definition of workplace health, beyond a traditional occupational safety and health (OSH) focus on physical exposure to harm: ‘A healthy workplace is one in which workers and managers collaborate to use a continual improvement process to protect and promote the health, safety and well-being of workers and the sustainability of the workplace’. [5] Key elements of desirable HWE include:

1. A focus on physical and mental health. [6]
2. A longer-term view on immediate and chronic health and safety risks. [7]
3. A focus on ‘psychosocial’ risks in workplace health. [8] Sources of risk relate to excessive workloads, poor managerial communication, limited worker involvement in making decisions, insecure work, and harassment and discrimination.
4. Greater emphasis on positive psychosocial measures to improve wellbeing, connected to high job satisfaction. [9,10]
5. Respectful and inclusive workplace decision-making processes. [10]
6. Recognition that boundaries between ‘work’ and ‘life’ are often blurry. [11] Private lives and natural and social environments have a clear impact on working lives.

This agenda may be driven by three different motivations. [5] First, it is ‘the right thing to do’. A safe and healthy workplace is ‘a fundamental human right’. Including workers in workplace decision-making is self-evidently valuable. Second, it is the ‘smart thing to do’. A healthy workforce is more productive. An included and engaged workforce contributes to continuous workplace innovation. Third, it is the ‘legal thing to do’. Companies must follow regulations to minimise workplace harms, including physical impacts and factors such as discrimination.

## 3 What policy instruments should be used to support HWE?

While this expanded agenda is welcome, it is incomplete until we can map these ambitions onto a *policy mix* consisting of multiple policy instruments that would contribute to its delivery and outcomes. To that end, first we build on the list of instruments identified by Vitrano and Micheli [12] as essential to OSH policies, including:

• Education to provide essential professional skills.
• Training at work, to improve skills and knowledge of safety and health.
• Guidelines for workplace practice and to encourage continuous improvement (backed by external research).
• Workplace awareness raising, and direct support for organisations.
• Monitoring health and safety, including reports on workplace risks and accidents.
• Surveillance to prevent injuries and diseases at work.
• Enforcement of regulations via official external inspections.
• Accreditation standards, such as creating benchmarks and best practices.
• Insurance coverage to mitigate company risk (if meeting OSH standards).
• Financial incentives or penalties used by governments to encourage or punish workplace behaviours.

Second, we draw on the expanded HWE agenda to identify the kinds of policy instruments that could be used to improve workplaces:

1. A broader definition of health.
• Policy coordination instruments: Integrate policies that focus separately on physical and mental health.
2. A longer-term view.
• Policy guidance and research: Anticipate and prevent the impact of workplaces or routine work on musculoskeletal disorders (MSDs) and poor mental health.
• Policy strategy and guidance: Seek long-term approaches to worker retention, to keep workers healthier and in work for longer.
3. A broader definition of workplace negative contributors to mental health.
• Policy regulation: Maintain manageable workloads to avoid stress and overwork.
• Policy regulation: Prevent workplace discrimination and harassment (including psychological, sexual, and racial).
• Policy guidance or regulation: Encourage positive measures, such as to support workers during pregnancy or parental leave.
4. A greater emphasis on positive psychosocial measures, including respectful and inclusive decision-making.
• Policy collaboration: Foster greater workplace autonomy where employees feel that they have a meaningful say in how they work and have discretion to manage competing demands and influence how the job is done (to improve job satisfaction and a sense of purpose).
• Policy collaboration: Ensure that workers feel part of decision-making, such as via trade union and management partnerships.
• Policy collaboration: Include workers in organisational change and innovation, to contribute to a vision for a healthy workplace and widen the distribution of skills relevant to an organisation's ability to deal with unexpected events.
• Policy guidance: Ensure that workers receive effective communication and support from management and colleagues.
6. A sense that boundaries between work and life are blurry.
• Policy coordination: connect multiple measures that address aspects such as caring responsibilities, dealing with family illness or loss, flexible and out-of-office working, and adaptations to climate change or other crises.

## 4 Who should be responsible for making and delivering HWE policy instruments?

To some extent, it is possible to map these policy instruments onto the organisations that *could* be key to influencing, making, or delivering each measure. For example, Table 1 maps Vitrano and Micheli's [12] list of instruments and the key actors who would be broadly responsible. However, even in this relatively clear context, there is uncertainty about OSH aims and responsibilities, producing different models across national contexts (e.g. Ref. [13] on contrasting implementation between Croatia and Portugal).

**Table 1:** Potential responsibilities for OSH policies.

OSH policies	Key bodies or policy actors
Education	Universities, national research centres
Training	Ministry of labour, insurance institutes and research centres, trade unions, employer associations, chartered institutions, EU-OSHA
Guidelines	EU-OSHA, ILO, national bodies, insurance, labour inspection authorities, SLIC (Senior Labour Inspectors Committee)
Awareness and Support	Labour inspection authorities, membership associations, EU-OSHA
Insurance	Insurance companies (public or private)
Financial incentives	Government bodies, labour inspection authorities, insurance companies
Health and Safety surveillance	Representatives, inspectors and committees
Monitoring health and safety	Statistical institutes, insurance institutes, labour inspection authorities, research centres
Enforcement of regulations/inspections	Labour inspection authority, local authorities
Accreditation standard	Institutes for standardisation
Research (academic-practical)	Research institutes, universities

The expanded policy agenda accentuates this difficulty of pinpointing responsibilities for HWE (Table 2). A broad statement of *possible responsibilities* exposes tensions regarding who *should take responsibility* for the success of HWE policies and the balance between *state intervention* to regulate behaviour and *encouragement* for businesses to innovate.

**Table 2:** Potential responsibilities for OSH and HWE policies.

Potential new HWE policy instruments	Potential key bodies or policy actors
Workplace-worker representation	Workers and trade unions, company support for workplace autonomy, National labour ministries
Training of OSH practitioners and inspectors	Associations of OSH practitioners, OSH departments in large organisations, SLIC
Dissemination of new knowledge	Industry associations, conferences, networks. EU-OSHA research and guidance, SLIC
Workload and working time	EU regulations, member state legislation
Anti-discrimination and positive support	EU regulations, member state legislation on discrimination and harassment. Workplace support for pregnancy, parental leave, workers with caring responsibilities, workplace redesign.
Medical and health interventions to connect physical and mental health	Ministries of health, hospitals, rehabilitations centres (e.g. health promotion and support; return to work or managing absence)
Wider work-life factors, including social, economic and environmental factors	EU agencies, member states, subnational authorities, employers (e.g. member state social security rules; subnational and employer support to adapt workplaces); trade unions.

Our research on EU policy shows that this lack of clarity on HWE responsibilities undermines a clear sense of purpose and progress. In particular, we find a disconnect between two intersecting approaches to taking responsibility for HWE. First, the EU takes a *top-down* approach to regulations to oblige and enforce minimum OSH standards, backed by external inspection, monitoring, guidance, and benchmarks to articulate key standards and reduce variations in national inspection and sanction practices e.g. Refs. [9,[14], [15], [16]] Second, it takes a *bottom-up* approach to going beyond minimum standards, such as to issue EU-OSHA guidance on HWE innovation and good practice. In other words, while EU OSH policy is backed by regulation and enforcement, the EU HWE agenda focuses primarily on encouraging employers and managers to collaborate with workers to improve workplace practices.

We find an overall effect – of top-down and bottom-up messages – that may be disappointing to audiences who expect the call for a ‘fundamental shift’ towards HWE to translate into substantive outcomes. We find a rhetorically ambitious strategy, but without the clear means to produce collaborative public and private action that is consistent with this ambition. The EU devotes considerable energy to OSH regulation and encouraging national and organisational compliance but uses networking and guidance tools to encourage organisations to support healthy working environments. Further, relatively few companies are motivated and able to innovate, such as the large companies, in export-oriented and technology-based sectors which depend on the retention of skilled labour, innovation and their reputations as good employers [17]. Such companies may be able to demonstrate what innovative and maximal approaches to HWE look like, but most others may not have the means or motivation to learn or emulate.

## 5 Discussion: A cautionary tale for healthy workplace advocates

Although we describe modest policy progress, the EU is at the forefront of worker health and safety globally and its (and key member state) experiences offer far wider international lessons. Therefore, by focusing on the EU, we seek to prompt wider debate by highlighting broader unresolved questions: what exactly does HWE policy mean in theory and practice, and what would a successful HWE approach look like? Then, rather than asking policy actors to choose between a top-down or bottom-up model for HWE success, we expect both approaches to endure in some form, which should prompt policy actors to clarify how they can secure the best of both worlds. A *top-down* approach helps to oblige and enforce minimum OSH standards, but policymakers seem reluctant to raise the minimum when facing employer pushback. A voluntary, guidance-informed, *bottom-up* approach to going beyond minimal OSH standards seems destined to prompt useful but limited HWE innovation. The result may be meaningful HWE practices in a small number of leading organisations, with limited support for policy learning and emulation by other organisations.

Overall, we provide a cautionary tale in which HWE outcomes remain a long distance from the fundamental shift envisaged in the special issue of this journal. Such analysis provides a reminder that ‘the implications for multi-disciplinary public health practice are clear’ but the likely impact on policy and workplace practice is not. To make more tangible progress, healthy workplace advocates need to get from vague and self-evident aspirations towards defining exactly what HWE is, what instruments are essential, and who would be responsible for their design, delivery, and coordination.

## Ethical statement

The authors received ethical approval from the University of Stirling General University Ethics Panel (15.4.25) for the interview research contributing to the WAge project (although we do not report interview data in this Policy and Debate article)

## Funding

The WAge project (Healthy working environments for all ages: An evidence-driven framework) is funded by the Horizon Europe Framework Programme (101137207). WAge is developing a tool to help employers support the long-term physical and mental health of workers (this paper draws on our research for Deliverables 1.5 and 1.6). The WISEWORK consortium contains projects to support preventive, compassionate, sustainable, and well-designed workplaces. [18]

## Declaration of competing interest

The authors declare the following financial interests/personal relationships which may be considered as potential competing interests: None.
